# Aspirin use and bleeding volume in skin cancer patients undergoing surgery: a randomized controlled trial

**DOI:** 10.1186/s40199-016-0159-4

**Published:** 2016-07-28

**Authors:** Arman Engheta, Shahryar Hadadi Abianeh, Ali Atri, Mehdi Sanatkarfar

**Affiliations:** 1 Department of Plastic Surgery, Imam Khomeini Hospital, Tehran University of Medical Sciences, Tehran, Iran; 2 Department of Plastic Surgery, Razi Hospital, Tehran University of Medical Sciences, Vahdat Eslami st, Tehran, Iran; 3 Department of Anesthesiology, Razi Hospital, Tehran University of Medical Sciences, Tehran, Iran

**Keywords:** Acetyl salicylic acid, Skin cancer, Surgery, Bleeding, Complication, Aspirin

## Abstract

**ᅟ:**

We investigated the occurrence of bleeding complications in patients who underwent skin tumor surgery and compared it between Aspirin users and a placebo control group. In this double blind randomized controlled trial, 32 patients who continued taking aspirin (intervention group) and 38 patients who stopped taking Aspirin (placebo group) before surgery were compared in terms of intraoprative and postoperative bleeding problems, hematoma and local signs of coagulopathy. There was no statistically significant difference in intraoprative bleeding between the study groups (*P* = 0.107). We concluded that continuation of Aspirin therapy had no significant effect on bleeding complications in patients who underwent skin tumor surgery.

**Trial registration:**

IRCT201602049768N5

**Graphical abstract:**

Flow chart of the study process and its final finding
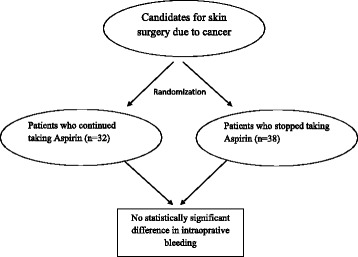

Discontinuation of anticoagulant or anti platelet agents before skin surgery is still a challenge due to the lack of proper recommendations in the current guidelines [1]. For the decision making, the surgeon should consider several patient-related factors, such as indication of the treatment, patient’s condition and the underlying disease, in order to decide about the continuation or interruption of the drug [2, 3]. Skin surgeries are considered as one of the safest and simplest surgeries. However, rapid increase in the use and new indications of anticoagulant drugs, particularly aspirin, requires specific attention toward their use in skin surgeries [4, 5].

However, the evidence regarding the continuation or discontinuation of Aspirin before skin surgery is inconsistent. In the present study, we aimed to monitor the bleeding complications in patients who underwent skin tumor surgery and compared it between Aspirin users and a placebo control group.

In this double-blind randomized controlled trial, we enrolled patients with non-bleeding skin tumors who were under treatment with aspirin due to any indication. The inclusion criteria were use of Aspirin for at least 3 months before surgery with a daily dose of 80 mg, age between 40 and 75 years, giving an informed consent for taking part in the study, and international normalized ratio (INR) of 1–1.5. Our exclusion criteria included as follows; Having dementia, movement disorder, simultaneous participation in another trial, patients with life-threatening cardiovascular diseases (i.e. New York Heart Association class III or more, history of previous myocardial infarction, severe heart valve disease), bleeding disorders, use of antiplatelets other than Aspirin or anticoagulants and positive history of gastrointestinal bleeding. Moreover, patients who did not follow the prescription rules, those who had a disease that required Aspirin discontinuation or Aspirin intolerance were also excluded. In order to make sure about the drug compliance of the patients, they were asked to bring the blister pack of the consumed tablets.

Using block randomization, patients were randomized into intervention and control groups, matched for age and sex. Both groups were asked to discontinue their Aspirin 7 days before the surgery and they received packed drugs of the trial including Aspirin (80 mg) for the intervention group and placebo for the control group.

Before operation, demographic and baseline clinical characteristics were collected from the patients. The clinical data included the presence of bleeding risk factors, type of skin tumor, number and size of the tumor(s), location of the lesion, drug history and blood test. For every patient, standard resection for the tumor was performed regarding its size and other clinical characteristics. Type of operation and data regarding anesthesia, cautery, need for osteotomy and other surgical characteristics were recorded for the patient. We measured the bleeding by weighing the dressing gases during and after operation up to 24 h. The nurse who was in charge of weighing the gases was blinded to the study protocol. Primary endpoint of the study was the amount of bleeding within and early after surgery. Secondary endpoints were need for early changing of the dressing, development of hematoma or local anticoagulation disorders such as petechia or ecchymosis.

Categorical variables were analyzed by the chi-square test. Continuous variables are presented as means ± standard deviation, or as median and interquartile ranges, as appropriate. Differences between groups in normally and non-normal continuous variables were assessed using the unpaired Student’s t test and the Mann–Whitney U test, respectively. All probability values were two-tailed and a *P*-value < 0.05 was considered significant. Data were analyzed with Statistical Package for the Social Sciences (SPSS) for Windows, version 15.0 (SPSS Inc., Chicago, Ilinois, United States of America).

In the present study, 38 patients were randomized to the intervention group and 38 patients were included in the control group. However, after randomization it was revealed that three patients had used antiplatelet or anticoagulant drugs and three other patients refused to continue the study; so, they were excluded from the final analysis (*n* = 32 for the intervention group). The frequency of diabetes and cardiovascular disease was significantly higher in the intervention group (*P* = 0.009 and *P* = 0.002, respectively). Details of the demographic and baseline clinical characteristics of the study groups are shown in Table 1.

**Table 1:** Comparison of the baseline characteristics between the study groups

Characteristic^a^	Intervention (*n* = 32)	Placebo (*n* = 38)	*P*-value^†^
Age, year	65.8 ± 2.3	64.1 ± 1.7	0.218
Male gender, *n* (%)	24 (75)	29 (76.3)	0.683
Diabetes, *n* (%)	13 (40.6)	5 (13.2)	0.009
Hypertension, *n* (%)	23 (71.9)	19 (50)	0.063
Cardiovascular diseases, *n* (%)	21 (65.6)	11 (28.9)	0.002
Smoking, *n* (%)	4 (12.5)	5 (13.2)	0.999
Opium abuse, *n* (%)	3 (9.4)	4 (10.5)	0.999
FBS, mg/dl	123.9 ± 58.0	121.7 ± 44.9	0.696
BUN, mg/dl	37.3 ± 8.9	33.7 ± 8.7	0.064
Creatinine, mg/dl	0.94 ± 0.24	0.95 ± 0.25	0.723
Hemoglobin, g/dl	13.7 ± 1.3	14.9 ± 4.4	0.054
Platelet, 1/mm^3^	208.4 ± 81.9	200.3 ± 45.1	0.925
INR	2.1 ± 4.7	1 ± 0.01	0.096
PT, sec	13.3 ± 3.2	12.9 ± 2.3	0.001
PTT, sec	28.1 ± 4.1	28.9 ± 3.2	0.114
CT, sec	327.3 ± 72.2	321.7 ± 62.9	0.669
BT, sec	152.7 ± 59.7	148.7 ± 44.6	0.791

*BT* Bleeding time, *BUN* Blood urea nitrogen, *CT* clotting time, *FBS* Fasting blood sugar, *INR* International normalized ratio, *PT* Prothrombin time, *PTT* Partial thromboplastin time

^a^Variables are shown as mean ± standard deviation or frequency (percentage) where appropriate

†*P* < 0.05 was considered as statistically significant

Based on the pathology report, characteristics of the tumors and operation were comparable between the two groups as shown in Table 2.

**Table 2:** Comparison of the tumoral and operative characteristics between the study groups

Characteristic^a^	Intervention (*n* = 32)	Placebo (*n* = 38)	*P*-value^†^
Location			0.908
Face	10 (29.4)	13 (30.9)	
Nose	6 (17.6)	4 (9.5)	
Ear	4 (11.7)	5 (11.9)	
Neck	0 (0)	1 92.3)	
Scalp	9 (26.4)	14 (33.3)	
Other	5 (14.7)	5 (11.9)	
Type			0.675
Basal cell carcinoma	24 (75)	32 (84.2)	
Squamous cell carcinoma	5 (15.6)	5 (13.2)	
Melanoma	1 (3.1)	0 (0)	
Not reported	2 (6.2)	1 (2.6)	
Size of lesion			0.17
< 3 cm	15 (46.9)	14 (35)	
3–6 cm	15 (46.9)	24 (60)	
> 6 cm	1 (3.1)	2 (5)	
Not reported	1 (3.1)	0 (0)	
Number of lesions			0.478
1 lesion	22 (68.8)	29 (76.3)	
2 lesions	3 (9.4)	6 (15.8)	
3 lesions	2 (6.2)	1 (2.6)	
4 lesions and more	3 (9.4)	1 (2.6)	
Not reported	2 (6.2)	1 (2.6)	
Type of surgery			0.72
Flap	24 (70.6)	28 (68.3)	
Graft	9 (56.4)	13 (31.7)	
Other	1 (2.9)	0 (0)	
Type of anesthesia			0.999
Sedative	31 (96.9)	38 (100)	
Not reported	1 (3.1)	0 (0)	
Cautery			0.999
Monopolar	31 (96.9)	37 (97.4)	
Bipolar	1 (3.1)	1 (2.6)	
Need for osteotomy	0 (0)	1 (2.6)	
Volume of bleeding, ml	30 [20, 80]	30 [17, 40]	0.107

^a^Variables are shown as frequency (percentage) or median [interquartile range] where appropriate

†*P* < 0.05 was considered as statistically significant

Bleeding in all participants was restricted to the operation time and none of the participants had postoperative bleeding. Median volume of bleeding was 30 gram in both groups (*P* = 0.107) (Table 2). None of the patients required early change of wound dressing and we observed no case of hematoma or local coagulation disorder.

We found no significant difference between patients who used Aspirin perioperatively and those who discontinued it beforehand. This finding is in line with similar previous studies [6–8], while the strength of our study is its randomized controlled trial design and its uniform population that consisted of skin cancer patients. We also observed no complication within the study period.

Based on our findings, perioperative Aspirin therapy had no significant effect on bleeding complications in patients who underwent skin tumor surgery. Currently, surgical bleedings can be controlled easily by electrocauterization and are not potentially life-threatening. It seems that dermasurgeons should be more informed about the safety of Aspirin use in skin surgeries based on the current body of knowledge. Larger studies can also contribute to the elucidation of the use of multiple antiplatelet and anticoagulant agents during skin surgeries.

## Abbreviations

BT: Bleeding time
BUN: Blood urea nitrogen
CT: Clotting time
FBS: Fasting blood sugar
INR: International normalized ratio
PT: Prothrombin time
PTT: Partial thromboplastin time
SPSS: Statistical Package for the Social Sciences
